# Polyclonal evolution of Fanconi anemia to MDS and AML revealed at single cell resolution

**DOI:** 10.1186/s40164-022-00319-5

**Published:** 2022-09-27

**Authors:** Lixian Chang, Zejia Cui, Deyang Shi, Yajing Chu, Bichen Wang, Yang Wan, Qiuyi Ma, Ranran Zhang, Haoyuan Li, Xuelian Cheng, Tao Cheng, Xiaofan Zhu, Cheng Li, Weiping Yuan

**Affiliations:** 1 State Key Laboratory of Experimental Hematology, National Clinical Research Center for Blood Diseases, Haihe Laboratory of Cell Ecosystem, Institute of Hematology & Blood Diseases Hospital, Chinese Academy of Medical Sciences and Peking Union Medical College, Tianjin, 300020 China; 2 Joint Graduate Program of Peking-Tsinghua-NIBS, Academy for Advanced Interdisciplinary Studies, Peking University, Beijing, 100871 China; 3 School of Life Sciences, Center for Bioinformatics, Center for Statistical Science, Peking University, Beijing, 100871 China

**Keywords:** Fanconi anemia, Myelodysplastic syndromes, Acute myeloid leukemia, Clonal evolution

## Abstract

**Background:**

Fanconi anemia (FA) is a rare disease of bone marrow failure. FA patients are prone to develop myelodysplastic syndrome (MDS) and acute myeloid leukemia (AML). However, the molecular clonal evolution of the progression from FA to MDS/AML remains elusive.

**Methods:**

Herein, we performed a comprehensive genomic analysis using an FA patient (P1001) sample that transformed to MDS and subsequently AML, together with other three FA patient samples at the MDS stage.

**Results:**

Our finding showed the existence of polyclonal pattern in these cases at MDS stage. The clonal evolution analysis of FA case (P1001) showed the mutations of *UBASH3A*, *SF3B1*, *RUNX1* and *ASXL1* gradually appeared at the later stage of MDS, while the *IDH2* alteration become the dominant clone at the leukemia stage. Moreover, single-cell sequencing analyses further demonstrated a polyclonal pattern was present at either MDS or AML stages, whereas *IDH2* mutated cell clones appeared only at the leukemia stage.

**Conclusions:**

We thus propose a clonal evolution model from FA to MDS and AML for this patient. The results of our study on the clonal evolution and mutated genes of the progression of FA to AML are conducive to understanding the progression of the disease that still perplexes us.

**Supplementary Information:**

The online version contains supplementary material available at 10.1186/s40164-022-00319-5.

## Background

Fanconi anemia (FA, MIM 607,139) is a rare autosomal recessive, X-linked (FANCB) or autosomal dominant (FANCR/RAD51) bone marrow failure disease, which occurs at a rate of 1–5 cases per million [1]. FA is caused by mutations in any of the 23 genes that are known in the FA/BRCA DNA repair pathway [2–4]. These genes are associated with defects in the repair of inter-strand crosslinks (ICL) in DNA and the maintenance of genomic stability.

Clinically, FA is mainly characterized by developmental abnormalities, bone marrow failure (BMF), and predisposition to cancer [5]. BMF is the main characteristic of FA and generally occurs when patients are between 5 and 10 years old, and the risk of BMF exceeds 90% in FA patients who are 40 and older [6, 7]. FA patients are also prone to develop cancer, mainly acute myeloid leukemia (AML) and myelodysplastic syndrome (MDS). The relative risk of AML and MDS in FA patients is 48- and 6000- fold when compared with the incidence in the general population [8], and patients with FA or BMF are usually monitored by both cytogenetic and morphological examinations for the development of MDS or AML progression.

Distinct clone formation is an evolutionary ability that offers both benefits and drawbacks. Moreover, the clonal evolution has long been implicated as a risk factor for the development of many diseases, including MDS and other cancers [9, 10]. Studies have shown that the risk of developing MDS or AML within three years of observing of an abnormal clone in FA patients was roughly 1 in 3 (35%) in comparison with 1 in 30 (3.3%) for those without an identifiable clone [11, 12]. Cytogenetic findings revealed that the copy number changes of chromosomes 1, 3 or 7 are risk factors for AML development in FA. The 3-year risk of developing MDS or AML in patients with aberrations of chromosome 3 was 90% and 17%, respectively [13, 14], while the presence of clonal mosaicism increased the cancer risk and poor prognosis in FA patients [15]. Quantitative proteomic and metabolomic analyses showed an association between the need for nitrogenous bases upon impaired DNA damage response (DDR) in FA cells with a subsequent increase in purine metabolism, indicating a potential role in oncogenesis [16]. Proteomic profiling and bioinformatics analysis have identified 12 key proteins that may play important roles in the progression of FA to AML, of which high expression of HIST1H1D, HIST1H3A, PSME1, and THRAP3 was found to associate with a poor prognosis of AML [17]. Although there are some genomic and proteomic studies for the changes of FA progressing to MDS or AML, the specific molecular events of clonal evolution regarding MDS/AML progression of FA patients remains unknown, and importantly, most of the previous studies are carried out on population cells [11–15]. Considering the chimeric and polyclonal nature of FA and its disease progression, single-cell sequencing may more accurately reflect the molecular changes during the clonal evolution in FA patients. Here we performed comprehensive genomic analyses in an individual patient with FA transformed to MDS and subsequently AML (P1001), in conjunction with three additional FA patients in their MDS stage (P1002, P1003, and P1004). We found that the polyclonal pattern was present at either MDS or AML stages in Patient P1001 and the IDH2 mutation containing clone is the dominant clone at AML stage.

## Methods

### Patient samples

Peripheral blood (PB), bone marrow (BM), and folliculus pili samples were obtained from the patient P1001. Bone marrow and folliculus pili samples of the other three patients who converted from FA to MDS were derived from P1002, P1003, and P1004. For patient P1001, genomic DNA was extracted from MDS and AML bone marrow mononuclear cells, as well as from 5 types of blood cells (B cells (CD45^+^CD19^+^), T cells (CD45^+^CD3^+^), NK cells (CD45^+^CD16/56^+^), Monocytes (CD45^+^CD14^+^) and Granulocytes (CD45^+^CD10/33^+^CD14^−^) in FA stage, and from PB cells during MDS and AML stage. Genomic DNA extracted from folliculus pili samples were used as germline control for somatic variant calling. For patient P1002, P1003, and P1004, genomic DNA were extracted from whole bone marrow cells, and folliculus pili samples at MDS Stage. All Genomic DNA of whole BM, whole PB, BMMNCs, PBMNCs, sorted cells, and folliculus pili samples were extracted by DNeasy Blood and Tissue Kit (QIAGEN) according to the manufacturer’s protocol. All patients provided written consent on a protocol approved by the Clinical Research Ethics Committee of Institute of Hematology and Blood Diseases Hospital, Chinese Academy of Medical Sciences (HG2021007-EC-1).

### Enrichment and sorting of mononuclear cells

Whole BM and peripheral blood samples were obtained from patients during the stage of FA. Mononuclear cells (MNCs) of bone marrow and peripheral blood were enriched by Ficoll (Histopaque^®^-1077, Sigma) following manufacturer’s instructions. PBMNCs at the first diagnosis of patient P1001 were stained for 30 min on ice with antibodies: CD45, CD3, CD19, CD16/56, CD14, CD10/33 to isolate T cells (CD45^+^CD3^+^), B cells (CD45^+^CD19^+^), NK cells (CD45^+^CD16/56^+^), Monocytes (CD45^+^CD14^+^), Granulocytes (CD45^+^CD10/33^+^CD14^−^). Then cells were sorted on an Aria III flow cytometer. For single-cell sorting, we used Lin^−^CD34^+^CD38^−^ to enrich for hematopoietic stem cells. After staining of surface markers, single cells were directly sorted into a 96-well PCR plate using Aria III flow cytometer. A list of antibodies is provided in Additional file 1: Table S3.

### Whole genome sequencing

Whole-genome sequencing was performed on the FA, MDS, AML, and folliculus pili (control) samples. Paired-end sequencing was performed using the Illumina HiSeq platform with 150 bp read length. For all samples, 65 to 100 Gb of WGS sequence was generated, corresponding to 40 × to 50 × haploid coverage of the reference genome.

### Single cell-genome amplification

Single cell-genome amplification was performed with Discover-sc^®^ Single Cell WGA Kit (Vazyme) according to the manufacturer’s protocol, which uses a multiple displacement amplification (MDA)-based isothermal amplification system to achieve indiscriminate amplification of the whole genome using a single cell or micro-sample as a template [18]. Then products are directly used as input for targeted amplification.

### Single-cell targeted sequencing

After single cell-genome amplification, targeted sequencing was performed. For analysis of single-cell targeted sequencing data, we removed low quality reads by Trim Galore v0.4.4 using the default parameters. Therefore, the quality reads were mapped to hg38 with BWA v0.7.15 [19] and then variant calling with GATK v4.1.4.1 [20]. We manually confirmed each target mutation with the Integrative Genomics Viewer, and the mutations with covering at least 5 × and VAF > 5% were considered positive. Detailed methods are available in supplementary material methods. Our high-throughput datasets have been deposited to public GSA-human repository with the Accession Number [HRA002517].

## Results

### Case history for sequential samples

The clinical features of 4 patients that participated in this study are summarized in Table 1. Patient P1001 was initially diagnosed with Fanconi Anemia at aplastic anemia (AA) stage (FA-AA) carrying the mutation of FANCA (FANCA exon32 c.3163C > T; FANCA c.2222 + 1G > T splicing) at 10-years-age (in 2010). The patient was followed up again in 2016 and 2018 with a diagnosis of MDS. In comparison with the 2010 sample, the karyotype is different from the result of 2016 and 2018 with a gain of chromosomes 8. In 2019, the patient progressed to AML-M5. Patient P1001 and her family chose not to receive hematopoietic stem cell transplantation as a treatment, and she died at age of 20 in 2021 due to a splenic hemorrhage complication. FA patient samples of P1002, P1003 and P1004 were collected at MDS stage.

**Table 1:** Clinical characteristics of four FA patients

Patient	Sex	Age	Family history	Deformity	Blood Routine Test			Karyotype	Mutation Gene	Diagnose
					WBC	HGB	PLT			
P1001	Female	9	One sister with leukemia	Café au lait spots, micro-ophthalmia and abnormal thumbs	2.63	59	42	No	FANCA c.3163C > T; FANCA c.2222 + 1G > T splicing	FA-BMF
		16			3.32	58	52	46,XX,dup(1)(q12q32) [12]/46,idem,add(6)(p25) [8]		FA-MDS
		18			2.81	75	38	46,XX,dup(1)(q21q32)[4]/46,XX,dup(1)(q21q32),add(6)(p22)		FA-MDS
		19			3.93	28	339	47,XX,dup(1)(q21q32), + 8,inc [15]		FA-AML
P1002	Male	10	No	Microcephaly, café au lait spots, micro-ophthalmia	3.05	109	41	46, XY [9]	FANCD2 c.2021 + 5G > A splicing FANCD2 c.3707G > A SLX4 c.5177C > T	FA-MDS
P1003	Female	9	No	Left kidney is absent and the right kidney is fused	3.01	102	38	46, XX [9]	FANCA c.C1303T FANCA c.C3031T	FA-MDS
		13			2.44	90	43			
P1004	Female	5	No	Café au lait spots, abnormal thumbs of right hand	1.8	94	33	46, XX [13]	FANCL c.1000 T > C FANCL c.691C > T	FA-MDS

*FA-BMF* BMF stage of FA; *FA-MDS* MDS stage of FA; *FA-AML* AML stage of FA

### An increased mutation number during the progression from FA to MDS/AML

**Fig. 1:**
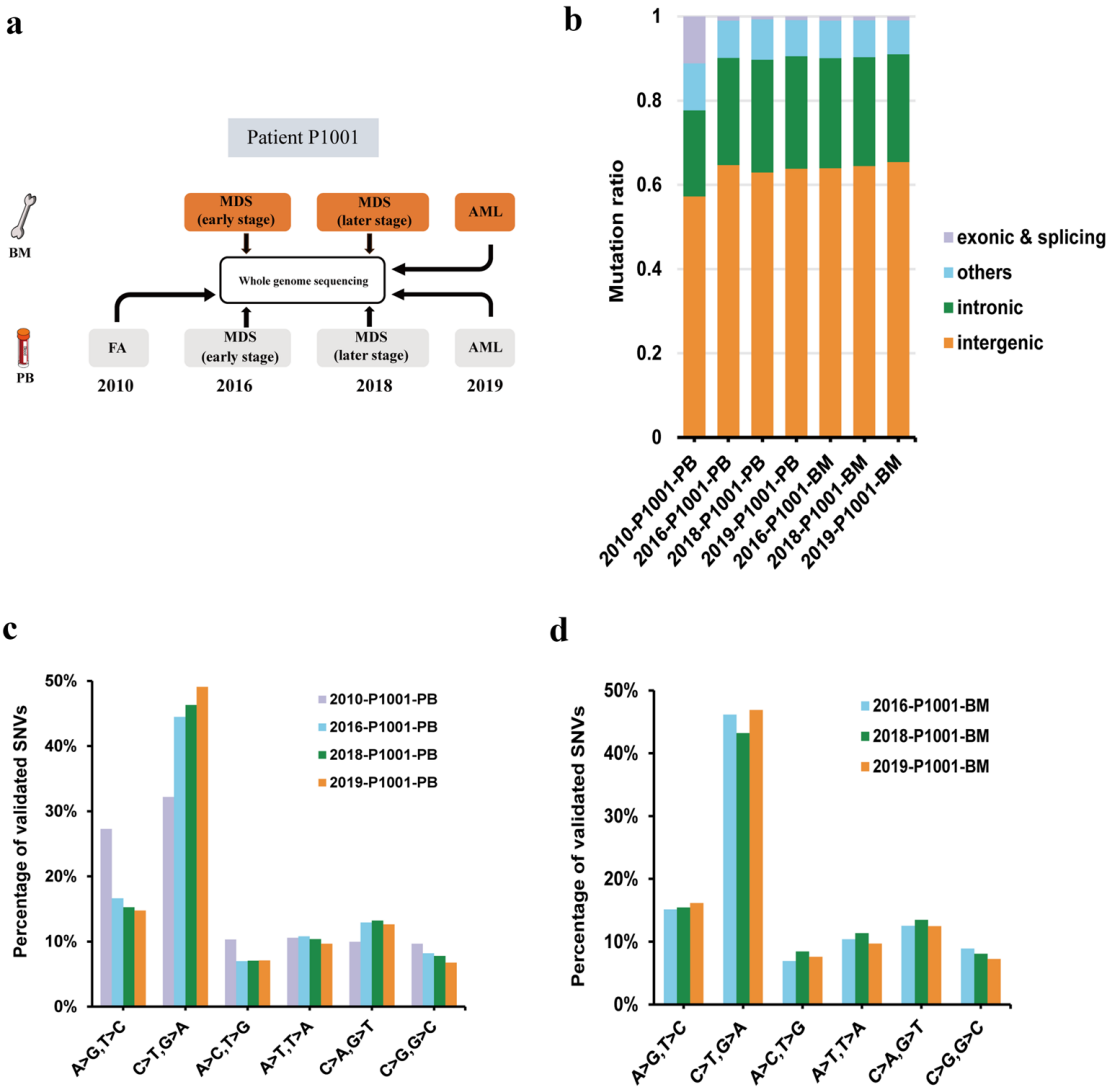
Somatic mutations quantified by whole genome sequencing in patient P1001 with FA who later progressed to MDS and sAML. **A** Schematics of experimental strategy of whole genome sequencing and single-cell validation of longitudinal, paired samples from patient P1001 with FA who later progressed to MDS and sAML. **B** Summary of mutations detected in peripheral blood and bone marrow samples from patient P1001 at different stages. **C** Spectrum of transition and transversion mutations in peripheral blood samples from patient P1001 in 2010, 2016, 2018, and 2019. **D** Spectrum of transition and transversion mutations in bone marrow samples from patient P1001 in 2016, 2018, and 2019

To analyze clonal evolution during the progression with FA-AA to MDS and AML in patient P1001, 4 peripheral blood samples of three stages (2010-FA, 2016-MDS, 2018-MDS and 2019-AML), 3 bone marrow samples of MDS and AML stages (2016-MDS, 2018-MDS and 2019-AML), and folliculus pili samples (as control samples) from the P1001 were obtained and subjected to whole-genome sequencing (Fig. 1a). In the FA-AA period of patient P1001, we obtained sequencing data for 5 types of blood cells in the peripheral blood (B cells, T cells, NK cells, mononuclear cells, and granulocytes) to analyze if mutations were shared across different cell populations and occured at the level of hematopoietic stem progenitor cells. The average number of single nucleotide variants (SNVs) and indels obtained at this stage is 1558, and the number of mutations in different types of blood cells was shown in Table 2. Interestingly, granulocytes have fewer exon and splicing mutations than other cells, possibly due to the fact that granulocytes develop and mature mainly in the bone marrow, had a short life span, and the trait of the patient with DNA damage repair deficiency [21]. However, we did not find overlapping non-synonymous mutations in the above mentioned 5 types of blood cells. An increase in the total number of mutations from 2016-MDS to 2018-MDS and to 2019-AML PB and BM samples was observed (Table 2). Notably, the proportion of mutations in the exon region is higher in FA-AA period than MDS or AML stage of the P1001, probably due to the instability of the genome (Fig. 1b). When the disease progressed to MDS and AML stages, the number of coding SNVs observed for each sample decreased within the range of coding mutations, and the accumulated  mutations were very likely caused by the mistakes during DNA replication in hematopoietic stem cells [22]. This finding was consistent with 2 genome-wide studies in MDS and AML published previously in which somatic SNVs are revealed to be passenger mutations that accumulate over time [22, 23]. Compared with the FA-AA period, the proportion of transversion mutations was significantly higher in MDS and AML (Fig. 1c, d). For the MDS and AML samples, the transversion and transition frequencies were similar to previously published results of MDS and AML [22, 24]. Our data indicated that total number of mutations is positively correlated with disease progression during the development of FA-AA into MDS and AML in patient P1001.

**Table 2:** Summary of somatic SNV and Indel mutations in patient P1001

Type	2010 - B	2010 - G	2010 - K	2010 - M	2010 - T	2016 - BM	2018 - BM	2019 - BM
Exonic and splicing	209	4	443	179	95	14	17	22
Intronic	299	285	290	322	377	367	478	595
Intergenic	808	817	871	938	979	898	1193	1521
Others	183	122	205	166	198	125	162	187
Overall	1499	1228	1809	1605	1649	1404	1850	2325

*B* B cells, *G* granulocyte, *K* NK cells, *M* mononuclear cells, *T* T cells

### Clonal architecture

**Fig. 2:**
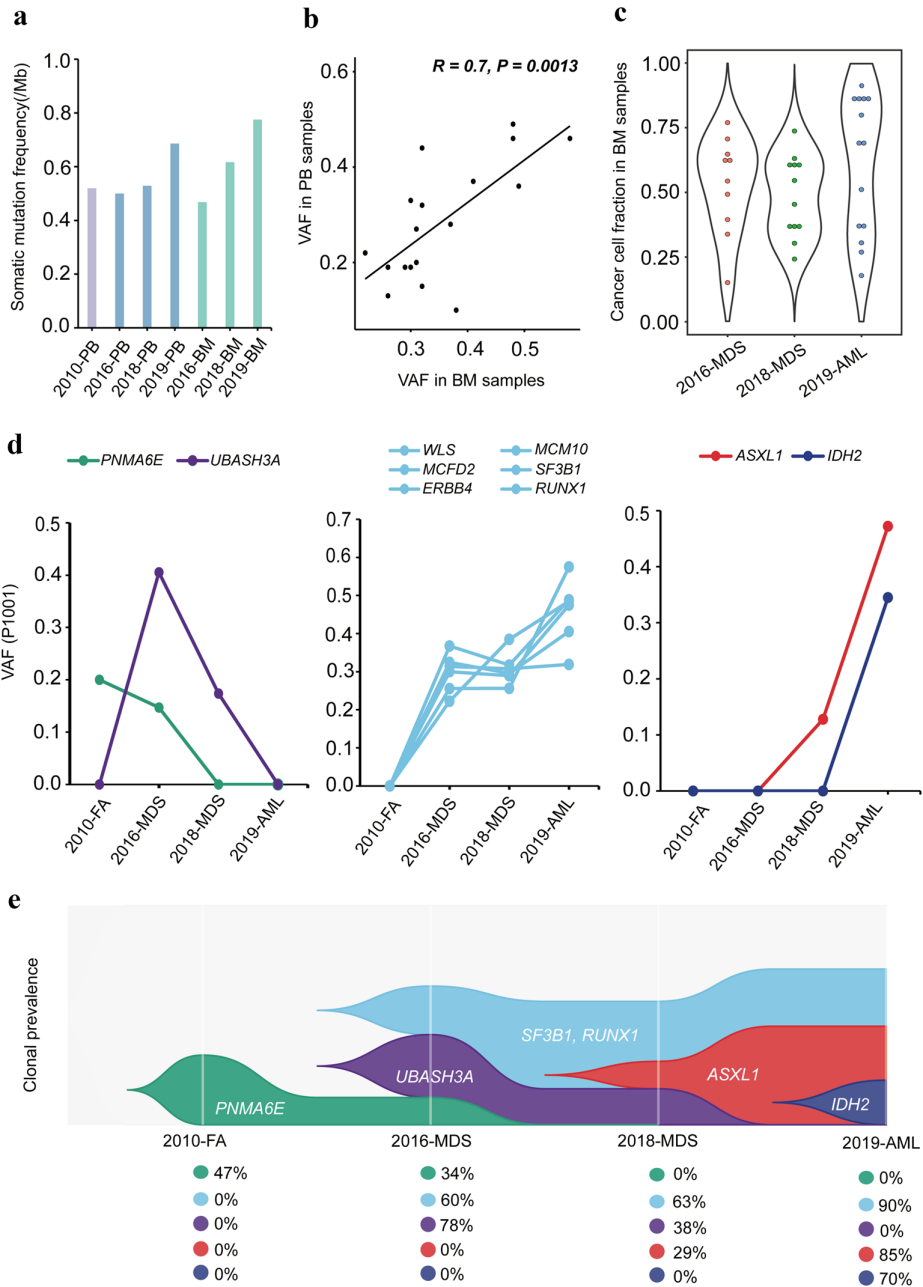
Schematic model of clonal evolution across patient P1001 disease progression. **A** Somatic mutation burden in peripheral blood and bone marrow samples from patient P1001 at different stages. **B** Correlation between variant allele frequency (VAF) in peripheral blood samples and bone marrow samples of patient P1001 (The Pearson correlation: 0.7; P = 0.0013). **C** Cellular prevalence in bone marrow samples of patient P1001. **D** Frequency of mutations in different clones from patient P1001 at different stages. **E** A model of clonal evolution based on the average value for the cellular prevalence in each cluster at each stage of progression

In order to explore the clonal evolution in patient P1001, we calculated the somatic mutation burden in peripheral blood and bone marrow samples from patient P1001 at different disease development stages, and we observed an elevation of mutation burden during progression from FA to AML (Fig. 2a). We also found a high correlation of the variant allele frequency (VAF) between PB and BM samples of patient P1001 (*P* = 0.0013; Fig. 2b), and the mutation results were shown in Additional file 1: Table S1. Therefore, the subsequent further analysis was based on the mutations that were identified in the patient’s BM samples. We calculated the cancer cell fraction within each BM sample of patient P1001, considering purity, ploidy and VAF as previously described [25]. We found that the trend in the frequencies of clonal mutations were consistent with changes in the somatic mutation burden of the patient. When the disease progressed to AML, there was an increase of the cellular prevalence (Fig. 2c). These results indicated that clonal complexity was higher in MDS and AML period than that of FA-AA stage.

To prospectively analyze clonal evolution during the progression of FA-AA to AML, we inferred the clonal architectures based on the VAFs of mutations. Five clonal groups were identified based on clusters of VAFs (Fig. 2d, e). The mutation in *PNMA6E* was frequently observed in the FA-AA stage, began to decrease as the disease progressed to the early stages of MDS, and disappeared in the late stage of MDS. In the early stage of MDS, the mutation of *UBASH3A* became a dominant clone, and the clone disappeared at the AML stage. In the same time frame, a clone including *WLS, MCM10, MCFD2, SF3B1, ERBB4*, and *RUNX1* mutations appeared at the early stage of MDS and increased with the progressive development of the disease. Among them, the *SF3B1* gene encodes subunit 1 of the splicing factor 3b protein complex, known to correlate with the prognosis of MDS [26]. When the disease progressed to the later stage of MDS and AML, new mutation clones (such as *ASXL1* and *IDH2)* emerged. The *ASXL1* is a member of the Polycomb group of proteins, and mutations in *ASXL1* were shown to be associated with myelodysplastic syndromes and chronic myelomonocytic leukemia [27]. *IDH2* is isocitrate dehydrogenase 2 and its mutations were reported to be associated with the pathogenesis of AML [28]. Based on the assumption that mutations with similar VAFs represent a clonal group as well as with unsupervised clustering methods, we demonstrated here that there was a founding clone in MDS and AML periods in patient P1001 and other specific subclones in each stage during the evolution of the disease sequentially from FA to MDS and AML. A graphic model of clonal progression is illustrated in Fig. 2e to demonstrate the evolutionary dynamics of clone across disease progression.

### Spatiotemporal subclonal evolution determined by single-cell targeted sequencing

**Fig. 3:**
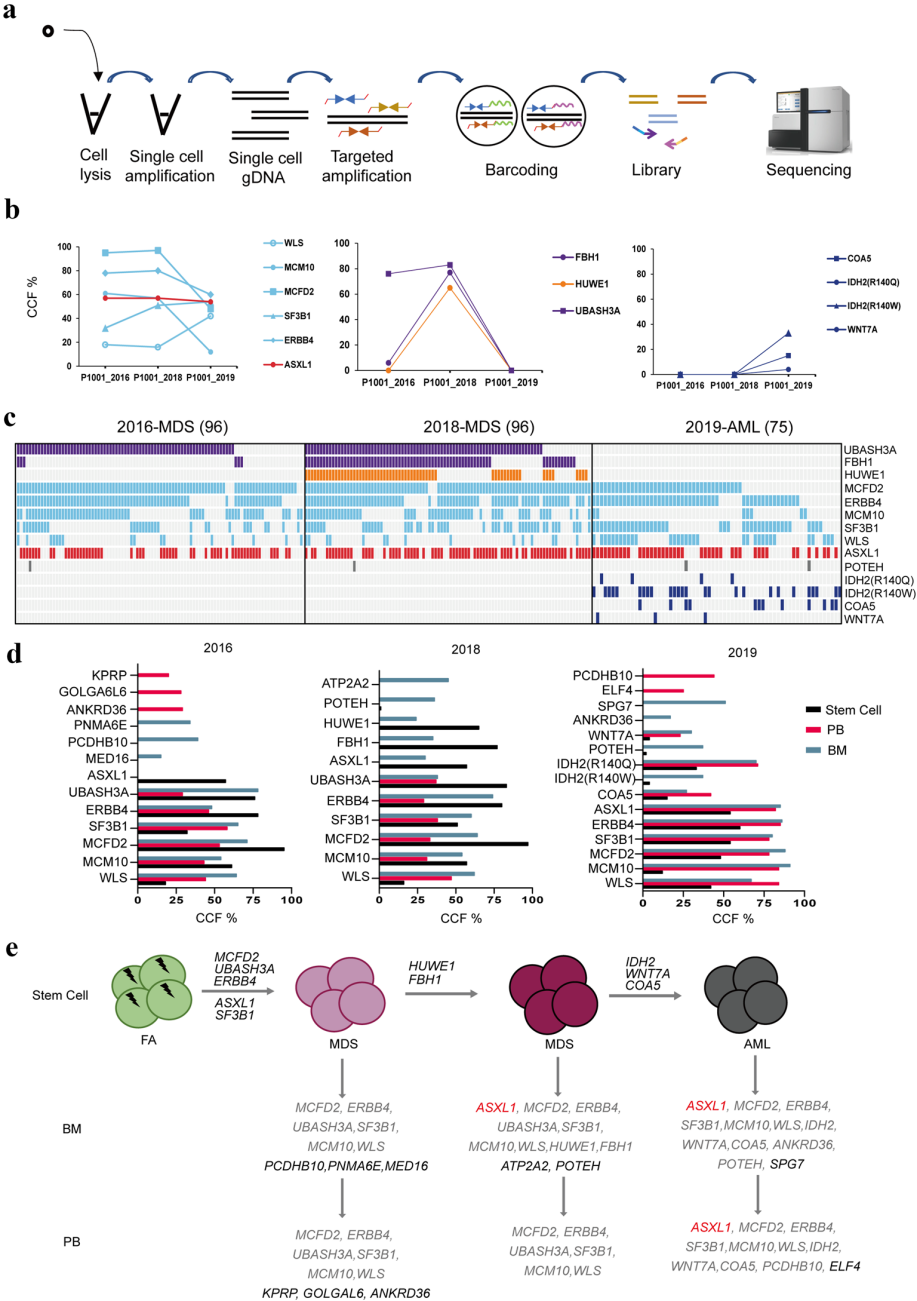
Spatiotemporal clonal evolution during the progression from MDS to AML determined by single-cell sequencing. **A** Experimental flow of single-cell targeted sequencing. **B** CCFs of shared (left), MDS-specific (middle), and AML-specific (right) mutations across all cell populations in patient P1001. **C** Single-cell targeted sequencing of mutations across different disease progression in patient P1001. Each column represents the sequencing results of one single cell at different stages, and the number of single cells tested in each stage is shown in parentheses. The occurrence of mutations in a same single clone is indicated by the same color as in (**B**). **D** CCFs of patient P1001 in stem cell, peripheral blood and bone marrow level. **E** Schematic model of clonal evolution in stem cell, peripheral blood and bone marrow level in patient P1001

To accurately identify clonal dynamics in different disease stages at stem cell level, we performed single-cell targeted sequencing of sorted stem cells with inferred mutations by whole genomic sequencing. The genes and specific primers are listed in Additional file 1: Table S2. We firstly performed single-cell DNA amplification with specific primers for targeted amplification, followed by single-cell barcoding and library construction (Fig. 3a). Targeted sequencing revealed that mutations of *ASXL1, ERBB4, MCFD2, MCM10, POTEH, SF3B1* and *WLS* were shared across all stages of the progression from MDS to AML (Fig. 3b). Interestingly, among them, the mutation in ASXL1 appeared only in stem cells, but not in whole BM and PB cells at year of 2016, suggesting that it had already presented at stem cell level with a high cellular prevalence before most cells in bone marrow and peripheral blood acquired the mutation. Additionally, *FBH1* mutations also emerged at the stem cell level in 2016 with a lower cellular prevalence as opposed to the whole genome sequencing data. By targeted sequencing of different stages of the disease samples, we also identified *FBH1, HUWE1* and *UBASH3A* as MDS-specific mutations, and *COA5, IDH2* (R140Q, R140W) and *WNT7A* as AML-specific mutations, respectively (Fig. 3b, c). Comparing mutated cancer cell fraction (CCF) of stem cells with that of bone marrow and peripheral blood, mutant clones differed in various cell populations, although they all shared the same sampling time (Fig. 3d). It suggests that different dominant clones may exist in different cell populations. Patients acquired *ASXL1* and *SF3B1* mutations at the level of stem cells in the early stage of MDS. In contrast, about 30% of PB cells carried the *ANKRD36* mutation that was not acquired by stem cells, and 40% of whole BM cells carried the *PCDHB10* mutation was also not acquired by stem cells and peripheral blood cells. In the later stage of MDS, while mutations *ATP2A2* and *POTEH* were not present in stem cells, they were found in whole BM cells. In AML populations, mutants of *IDH2* (R140Q, R140W) began to emerge, and the *CCF* of R140W mutant is high in both PB and BM samples, suggesting that mutation of *IDH2* may play a key role in the transition from MDS to AML. Meanwhile, *SPG7* and *ELF4* were specific mutations in whole bone marrow cells and peripheral blood cells, respectively (Fig. 3d).

Taken together, the results obtained by single-cell targeted sequencing led us to propose a FA patient-specific clonal evolution model in successive stages during the disease progression. In patient P1001, *UBASH3A* and *MCFD2* might act as founding clones and were acquired prior to the MDS stage, while mutations in *ASXL1* and *SF3B1* were gained at the early MDS stage. Acquisition of additional mutations in *HUWE1, FBH1* and *IDH2* might associate with the development of MDS and progression to AML, respectively (Fig. 3e).

To explore the relationship between somatic mutations and cell surface markers of leukemia stem cells, we combined single-cell targeted sequencing with FACS data. We found that the fraction of Lin^−^CD34^+^CD38^−^ in whole bone marrow cells increased during disease progression, in which the proportion of CD45RA positive cells became higher than in early disease stages. In a previous study, Lin^−^CD34^+^CD38^−^ cells (CD45RA^+^ and/or CD123^+^ and/or IL1RAP^+^) were found to be enriched for MDS-SC (MDS-stem cell) or AML-SC [29]. We thus sorted cells by CD45RA fluorescence intensity from high to low for analysis but failed to establish the relationship between single-cell mutational burden and changes in CD45RA fluorescence intensity.

### Cellular fraction of CNV gradually increased as the disease progressed

**Fig. 4:**
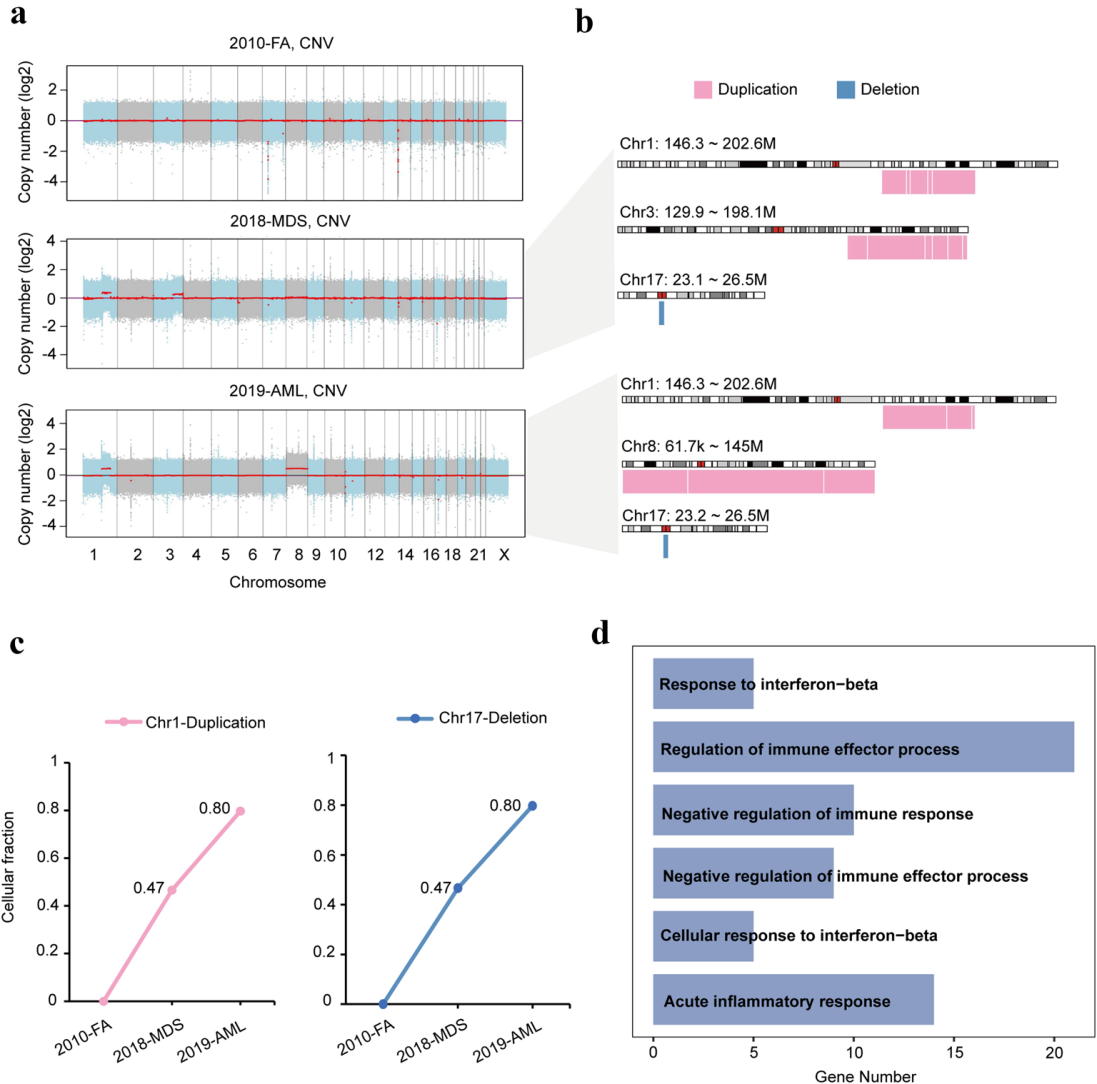
Copy number variants in patient P1001 with FA who later progressed to MDS and sAML. **A** The copy number variants of patient P1001 across disease progression of whole genome sequencing. **B** The copy number variants of patient P1001 during MDS and AML state. **C** The cellular fraction of the duplication and deletion in patient P1001 across disease progression. **D** The GO results of genes involved in the duplication on chromosome 1 of patient P1001

Study has shown that genomic instability of patients with Fanconi anemia, gain or loss of large fragments of chromosomes may affect the progression of the disease [30]. To clarify the diversification of CNV in the progress of FA-AA to MDS and AML, we further analyzed the CNV of patient P1001. Although CNV of the patient did not change significantly in the FA stage, an increase of approximately 60 Mb fragments and 70 Mb fragments of chromosome 1 and chromosome 3 was found at the MDS stage, respectively, consistent with the previous findings that chromosomes 1 and 3 were more frequently involved in clones in FA-AML patients than in de novo AML cases [31]. A decrease of chromosome 17 were also detected in the samples from the MDS stage. Interestingly, the same alterations in similar regions of chromosome 1 and chromosome 17 were also observed in AML samples developed from FA. Moreover, consistent with the patient's karyotype analysis, trisomy 8 was also detected at the AML stage (Fig. 4a, b). Previous studies showed that trisomy 8 is the most common numerical aberration in AML, and trisomy 8 positive AML is a heterogeneous group with the majority of cases containing additional cytogenetic aberrations [32, 33]. Further analysis of the cellular fraction of CNV found that duplication of chromosome 1 and deletion of chromosome 17 gradually increased as the disease progressed (Fig. 4c), indicating CNV may also be involved in the clonal evolution of the disease. To explore specific functions of CNV regions, gene ontology (GO) analysis of affected genes in chromosome 1 were performed (Fig. 4d). Genes regulating immune response and cellular response to interferon − β were significantly enriched, which was in line with previous studies that inflammation is involved in FA disease progression [34–38]. Our findings suggest that CNV may have an important impact on the progression of disease.

**Fig. 5:**
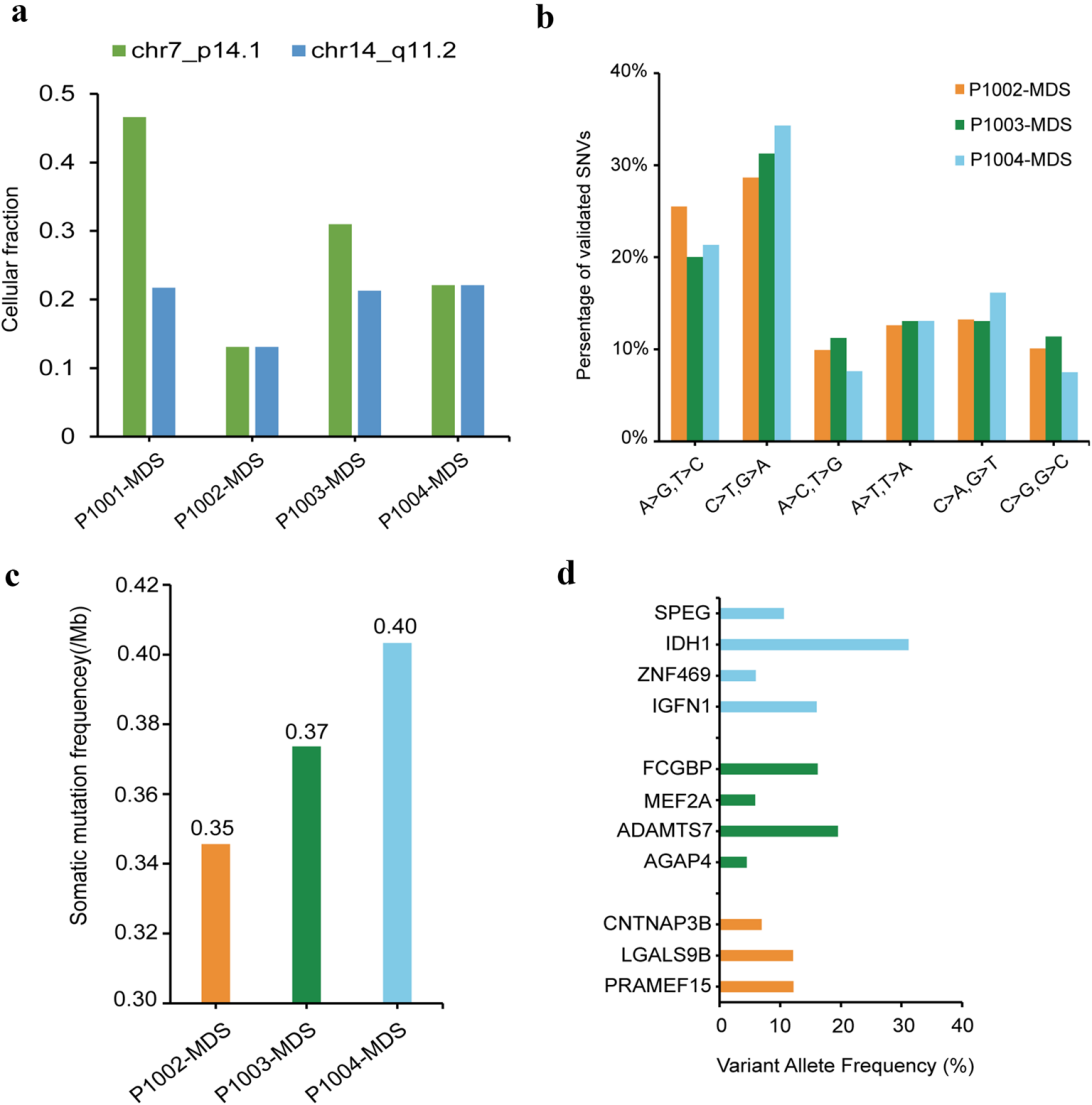
Copy number variants and mutations in FA-MDS patients. **A** Common copy number variants in the four FA-MDS patients. **B** Spectrum of transition and transversion mutations in bone marrow samples from patient P1002, P1003, and P1004. **C** The somatic mutation frequency of patient P1002, P1003, and P1004. **D** Mutation fractions of specific genes in patient P1002, P1003, and P1004

Cytogenetic abnormalities are frequently found in Fanconi anemia [30]. To further investigate the pattern of chromosomal and genomic abnormalities in FA and their association to MDS/AML, we compared cytogenetic abnormalities of 4 FA patients based on CNV data. Interestingly, loss of 7p14.1 and 14q11.2 were found in all patients at stage of MDS (Fig. 5a). GLI3, which localized on chromosome 7p14.1, was identified as a gene in which mutations cause Greig cephalopolysyndactyly (GCPS), a disease leading to craniofacial and limb mal-development [39]. Previous study also found significantly higher copy number variations at chromosome 7p14.1 and 14q11.2 in Dupuytren’s disease [40]. Furthermore, the decrease of cellular fraction in 14q11.2 at stage of AML of patient P1001 suggests that this region may not be directly related to disease progression, but rather is linked to abnormal limb development.

To determine the mutation burden and evolution process of FA patients that carry different mutant FA genes, we next examined the somatic mutation burdens of FA-MDS patients. BM samples from three different FA-MDS patients with mutations of *FANCA*, *FANCD2* and *FANCL*, respectively were used for whole genome sequencing. Consistent with patient P1001, an elevated percentage of C > T/G > A was found and is consistent with molecular change signatures in AML (Fig. 5b). However, somatic mutation frequency of these patients was slightly lower than P1001 at the MDS stage (Fig. 5c). Further exploration of the mutant genes in these patients revealed that different patients carrying the same or different mutant FA gene have different driver mutations (Fig. 5d). For patient P1004, *IDH1* might drive the progression of disease with its higher VAF, while *FCGBP* and *ADAMTS7* could expedite FA transformation to MDS for patient P1003 (Fig. 5d). Taken together, these observations demonstrated that patients who developed MDS-AML from FA had similar mutational patterns, with increased percentage of C > T/G > A, although the mutated genes and mutational burdens of these patients were quite distinct, indicating that there were significant heterogeneities among different patients during disease progression.

## Discussion

The molecular mechanism regarding the clonal evolution from AA to MDS and AML in FA patients remains elusive. Herein, a novel clonal evolution route was identified in patient P1001 with polyclonal pattern at both MDS and AML stages based on whole genome and single-cell sequencing analysis. The *UBASH3A*, *SF3B1*, *RUNX1* and *ASXL1* mutations gradually appeared and became the major clones at MDS stage, while *IDH2* gradually emerged as the dominant clone at leukemia stage. Additionally, our finding also showed the distinct bone marrow karyotypes and genetic alterations at MDS and AML. C > T and G > A transitions were the most abundant SNVs, which was in line with previous reports for AML cases [41]. Interestingly, in patient P1001, we observed that some of the mutations including *ASXL1* were detected at stem cell level at the early stage of MDS in the bone marrow, but appeared later in the peripheral blood in this patient. This may either due to that these mutations have lower VAF in PB than in BM at the current sequencing depths [42, 43], or the MDS-SC leading to the generation of MDS blasts could be different from those contributing to the progression to sAML [29].

In our study, a clone with *ASXL1* mutation was persistently detected throughout the disease progression although the mutation including clone appeared more apparent at late state of MDS. Interestingly, the clonal cells carrying *FBH1* and *HUWE1* mutations appeared at the later stage of MDS, whereas *UBASH3A, FBH1* and *HUWE1* mutations disappeared at AML stage. *ASXL1* is an epigenetic modulator and its mutation is frequently related to clonal hematopoiesis, MDS and AML [44–46]. In AML, *ASXL1* mutations were found to be are mutually exclusive to *DNMT3A, FLT3-ITD, NPM1* or *SF3B1* mutations. However, in our study, the FA patient carrying *ASXL1* mutation coexisted with the mutations of *IDH2* and *SF3B1*, probably reflected a unique character of FA patient due to impaired DNA repair pathway [47]. FBH1 helicase was reportedly to be associated with DNA homologous recombination repair process by regulating RAD51, and its abnormal function can lead to abnormal replication of DNA in cells [48, 49]. HUWE1 works as an important tumor suppressor through inhibiting the Ras-induced tumorigenesis [50]. At the MDS stage, there is an increased genetic mutation of DNA damage repair pathway within some cell clones, causing more apoptosis or death in related clonal cells. Nevertheless, once *IDH2* mutation was established, the clone containing *IDH2* mutation proliferated and prevailed in AML. Our single-cell sequencing data suggested continuous competition and disappearance of distinct clones during AML progression at MDS stage, which was supported by the sequencing data of other three MDS patients, showing highly heterogeneous mutations at the MDS stage. It is the mutation of genes related to disease progression acquired by clonal cells that determines how fast the progression of the disease.

**Fig. 6:**
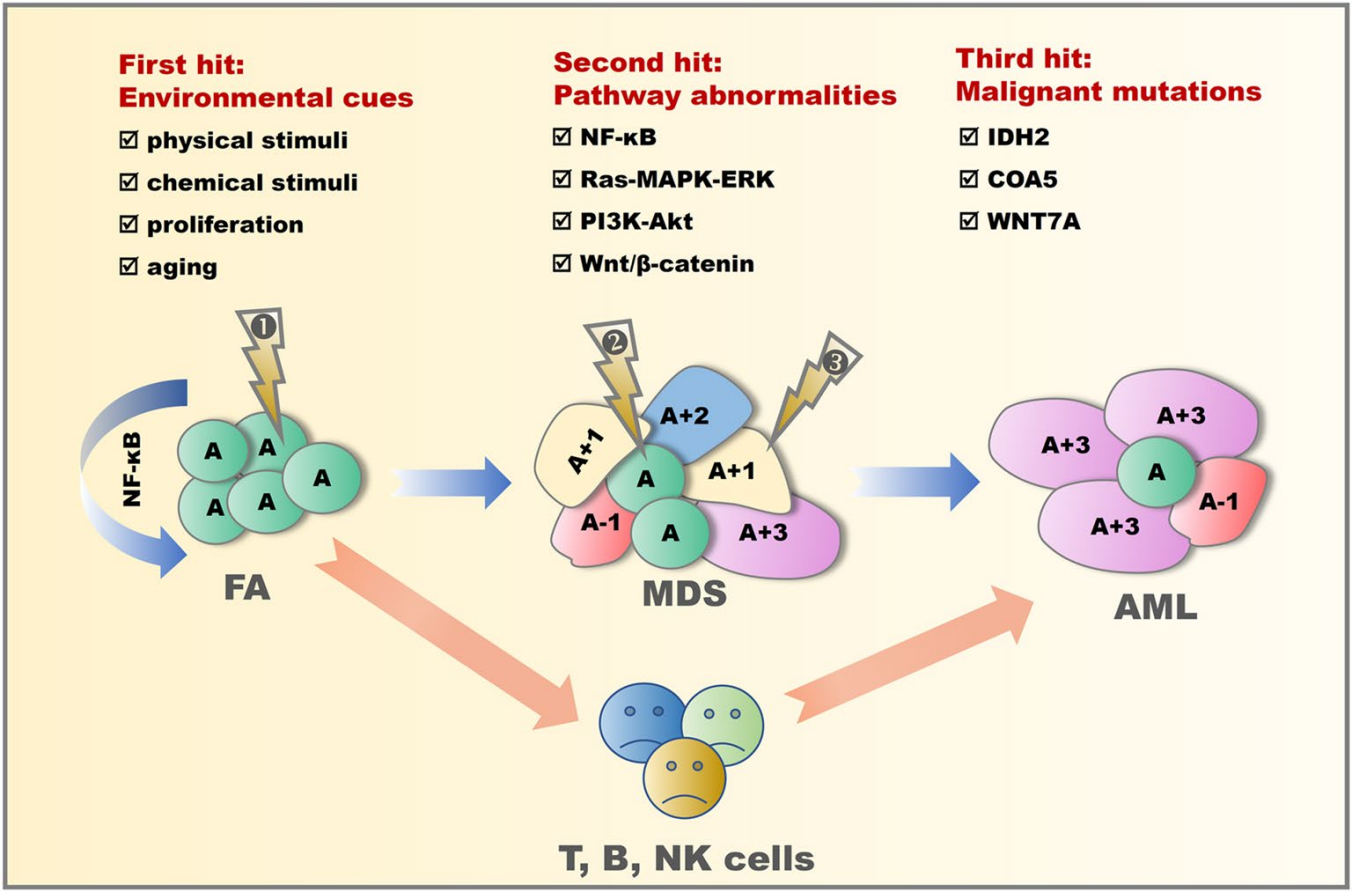
A proposed clonal evolution model. The cellular clonal evolution process of FA disease progression is a process in which multiple gene mutation clones compete for the survival or death under three progressive hits. The first hit is environmental cues such as physical and chemical stimulations, cell proliferation pressure and aging. The second hit is the aberrant activation of multiple cell signaling pathways. The third hit is the acquisition of tumor transforming mutations

Cells from FA patient may undergo multiple hits during the progression of FA in addition to their FA gene mutation (modeled in Fig. 6). As the first hit, multiple injuries such as physical, chemical, self-proliferative pressure and aging, cause increased somatic mutations and exacerbated genetic instability. The mutations cause hematopoietic stem cells to undergo apoptosis, leading to bone marrow failure and/or MDS. Next, a set of abnormal signaling pathways within cells serves as the second hit to contribute to further disease progression. For example, in this study, *UBASH3A, SF3B1, RUNX1, ERBB4* and *MCM10* are associated with NF-κB, Ras-MAPK-ERK, P13K-Akt and Wnt/β-catenin pathway abnormalities [51–59]. Activation of these signaling pathways can lead to accelerated cell proliferation, followed by an increased number of cells carrying genetic mutations. This is mainly presented in MDS disease state, during which some cells might die due to intolerance of such damage/changes. Acquisition of tumorogenesis-associated gene mutation may serve as the third hit, and cells acquired tumor characteristics might progress to AML. As we learned in this study, *IDH2* was the primary clone of AML, and the mutation of *IDH2* could be considered as the third hit of disease progression in patient P1001.

Notably, *IDH2* was shown to be associated with an improved prognosis in intermediate-risk AML patients [60]. Further, the *IDH2* mutations are closely linked to the mutations of *DNMT3A, SRSF2, NPM1, ASXL1* and *RUNX1*. Old adults with newly diagnosed *IDH2*-mutant AML benefited from Enasidenib, an inhibitor of *IDH2*-mutant protein [61]. Thus, IDH2 inhibitors such as Enasidenib may benefit patients with FA-related AML, and the use of such drug should be explored.

## Conclusions

In conclusion, we present a novel and dynamic model for the clonal evolution of FA disease progression using single cell sequencing with sequential samples from a single patient. With this model, we can determine which mutations are responsible for cells acquiring tumor forming characteristics when the disease progresses to AML. Moreover, our model provides insights into a molecular basis and treatment strategy for FA disease. With the ongoing expansion of sample size, our approach may identify more potential molecular targets for effective clinical treatment of FA and its progression.

## Supplementary Information

**Additional file 1: Table S1. **The mutation results in bone marrow and peripheral blood samples from patient P1001 at different stages. **Table S2. **Primers for single-cell targeted sequencing. **Table S3. **Antibodies for FACS experiments. **Material methods. **Including Whole genome analysis, Clonal analysis, and Single-cell targeted sequencing.

## Data Availability

All data generated or analyzed during this study are included in this published article and supplementary data. Our high-throughput datasets have been deposited to public GSA-human repository with the accession number [HRA002517].

## Abbreviations

FA: Fanconi anemia
MDS: Myelodysplastic syndromes
AML: Acute myeloid leukemia
B: B cells
G: Granulocytes
NK: Natural killer cells
M: Monocytes
T: T cells
ICL: Inter-strand crosslinks
BMF: Bone marrow failure
DDR: DNA damage response
PB: Peripheral blood
BM: Bone marrow
MNCs: Mononuclear cells
MDA: Multiple displacement amplification
GO: Gene ontology
GCPS: Greig cephalopolysyndactyly
SNV: Single nucleotide variants
FACS: Fluorescence activated cell sorting
