# Supplementary material for: Polyclonal evolution of Fanconi anemia to MDS and AML revealed at single cell resolution
Source: Exp Hematol Oncol. 2022 Sep 27;11:64. doi: 10.1186/s40164-022-00319-5 (PMC9513989; doi:10.1186/s40164-022-00319-5)
Supplement: Supplementary file 1 — Additional file 1: Table S1. The mutation results in bone marrow and peripheral blood samples from patient P1001 at different stages. Table S2. Primers for single-cell targeted sequencing. Table S3. Antibodies for FACS experiments. Material methods. Including Whole genome analysis, Clonal analysis, and Single-cell targeted sequencing. [file 40164_2022_319_MOESM1_ESM.pdf]

Table S1 The mutation results in bone marrow and peripheral blood samples from patient P1001 at different stages.

BM data

2016-MDS						
**CHROM**	**POS**	**REF**	**ALT**	**GeneName**	**Function**	**VAF**
chr1	68155160	G	C	WLS	nonsynonymous SNV	0.31
chr10	13204276	C	T	MCM10	nonsynonymous SNV	0.26
chr2	46907916	G	A	MCFD2	nonsynonymous SNV	0.37
chr2	1.97E+08	T	C	SF3B1	nonsynonymous SNV	0.32
chr2	2.11E+08	C	T	ERBB4	nonsynonymous SNV	0.22
chr21	42434851	C	A	UBASH3A	nonsynonymous SNV	0.41
chr5	1.41E+08	T	G	PCDHB10	nonsynonymous SNV	0.17
chrX	1.53E+08	TG	GC	PNMA6E	nonframeshift substitution	0.15
chr21	34886837	CAGTA	C	RUNX1	splicing	0.30

2018-MDS						
**CHROM**	**POS**	**REF**	**ALT**	**GeneName**	**Function**	**VAF**
chr1	68155160	G	C	WLS	nonsynonymous SNV	0.31
chr10	5903174	G	T	FBH1	nonsynonymous SNV	0.16
chr10	13204276	C	T	MCM10	nonsynonymous SNV	0.26
chr12	1.1E+08	T	G	ATP2A2	nonsynonymous SNV	0.21
chr2	46907916	G	A	MCFD2	nonsynonymous SNV	0.32
chr2	1.97E+08	T	C	SF3B1	nonsynonymous SNV	0.30
chr2	2.11E+08	C	T	ERBB4	nonsynonymous SNV	0.38
chr20	32435197	C	T	ASXL1	stopgain	0.13
chr21	42434851	C	A	UBASH3A	nonsynonymous SNV	0.17
chr22	15700206	G	A	POTEH	nonsynonymous SNV	0.16
chrX	53545140	C	T	HUWE1	nonsynonymous SNV	0.10
chr21	34886837	CAGTA	C	RUNX1	splicing	0.29

2019-AML						
**CHROM**	**POS**	**REF**	**ALT**	**GeneName**	**Function**	**VAF**
chr1	68155160	G	C	WLS	nonsynonymous SNV	0.32
chr10	13204276	C	T	MCM10	nonsynonymous SNV	0.58
chr15	90088702	C	T	IDH2	nonsynonymous SNV	0.17
chr15	90088703	G	A	IDH2	nonsynonymous SNV	0.34
chr16	89532536	T	G	SPG7	nonsynonymous SNV	0.24
chr2	46907916	G	A	MCFD2	nonsynonymous SNV	0.49
chr2	97208004	G	C	ANKRD36	nonsynonymous SNV	0.07
chr2	98600789	T	G	COA5	nonsynonymous SNV	0.12
chr2	1.97E+08	T	C	SF3B1	nonsynonymous SNV	0.41
chr2	2.11E+08	C	T	ERBB4	nonsynonymous SNV	0.48
chr20	32435197	C	T	ASXL1	stopgain	0.47

chr22	15700206	G	A	POTEH	nonsynonymous SNV	0.17
chr3	13819117	C	T	WNT7A	nonsynonymous SNV	0.14
chr21	34886837	CAGTA	C	RUNX1	splicing	0.48

Blood data

2016-MDS						
**CHROM**	**POS**	**REF**	**ALT**	**GeneName**	**Function**	**VAF**
chr1	68155160	G	C	WLS	nonsynonymous SNV	0.20
chr1	1.53E+08	G	A	KPRP	nonsynonymous SNV	0.08
chr10	13204276	C	T	MCM10	nonsynonymous SNV	0.19
chr15	20534261	A	G	GOLGA6L6	stoploss	0.12
chr2	46907916	G	A	MCFD2	nonsynonymous SNV	0.28
chr2	97208001	A	G	ANKRD36	nonsynonymous SNV	0.13
chr2	97208004	G	C	ANKRD36	nonsynonymous SNV	0.12
chr2	1.97E+08	T	C	SF3B1	nonsynonymous SNV	0.32
chr2	2.11E+08	C	T	ERBB4	nonsynonymous SNV	0.22
chr21	42434851	C	A	UBASH3A	nonsynonymous SNV	0.12
chr21	34886837	CAGTA	C	RUNX1	splicing	0.33
**2018-MDS**						
**CHROM**	**POS**	**REF**	**ALT**	**GeneName**	**Function**	**VAF**
chr1	68155160	G	C	WLS	nonsynonymous SNV	0.27
chr10	13204276	C	T	MCM10	nonsynonymous SNV	0.13
chr2	46907916	G	A	MCFD2	nonsynonymous SNV	0.15
chr2	1.97E+08	T	C	SF3B1	nonsynonymous SNV	0.19
chr2	2.11E+08	C	T	ERBB4	nonsynonymous SNV	0.10
chr21	42434851	C	A	UBASH3A	nonsynonymous SNV	0.18
chr21	34886837	CAGTA	C	RUNX1	splicing	0.19
**2019-AML**						
**CHROM**	**POS**	**REF**	**ALT**	**GeneName**	**Function**	**VAF**
chr1	68155160	G	C	WLS	nonsynonymous SNV	0.44
chr10	13204276	C	T	MCM10	nonsynonymous SNV	0.46
chr15	90088703	G	A	IDH2	nonsynonymous SNV	0.33
chr2	46907916	G	A	MCFD2	nonsynonymous SNV	0.36
chr2	98600789	T	G	COA5	nonsynonymous SNV	0.20
chr2	1.97E+08	T	C	SF3B1	nonsynonymous SNV	0.37
chr2	2.11E+08	C	T	ERBB4	nonsynonymous SNV	0.46
chr20	32435197	C	T	ASXL1	stopgain	0.43
chr3	13819117	C	T	WNT7A	nonsynonymous SNV	0.09
chr5	1.41E+08	T	G	PCDHB10	nonsynonymous SNV	0.21
chrX	1.3E+08	G	C	ELF4	nonsynonymous SNV	0.10
chr21	34886837	CAGTA	C	RUNX1	splicing	0.49

Table S2 Primers for single-cell targeted sequencing

Symbol	Primer sequence	Final concentration
ASXL1	TGATGATGAGGAGCAAGGA<br>AGGATTCAGGTGTGGAACT	1.5 μM
ANKRD36	ATTCCATTCAGGCTACAAGT<br>CATCAGCATCACCCAAGA	1.5 μM
ATP2A2	GAACCCTCCCACAAGTCT<br>GTAGCCTGAGTCACCTGTA	2.0 μM
COA5	TCTTGGTAGTTCTGATGTCA<br>TCTTCCTCTGAATCTTGCC	1.0 μM
COA5	CATGCGGTGATACCCTTACCA<br>AGGGGAAATCTGGTCCAACC	1.5 μM
ERBB4	TGTCTCGCATAGGAGTCAT<br>CAACTAGCACAATTCCAGAAG	2.0 μM
FBH1	TTAAGCGGAAGCATCTTACT<br>ACTCACAGCAGCAACATT	1.5 μM
FBH1	TGGCTAGCTTGTAGAATCGGT<br>GGACCCACACACTACAGGTTT	1.5 μM
HUWE1	TCTGAACACTTGCTGACTC<br>AGCCATCTTCCAGAATATCC	1.0 μM
MCFD2	CCTGAACCTGACTGTTGATT<br>GCCAGACCTACCTCCTTAT	1.0 μM
MCM10	CGCCTATACCCACTTCAAG<br>GGAACAGAATCACAGACTCT	1.5 μM
MCM10	GTCATGGAGCAGATTGCCCT<br>CAGTGCTTGTTCGGGAGTCT	1.5 μM
PCDHB10	CTCGTGGTGCTTGTCAAG<br>AGGAGGAAGAGCGAAGAC	1.5 μM
POTEH	CAGCCTTCTACTTGAGCAA<br>CCTGAACTGAACTATGACATC	1.5 μM
POTEH	GCTGTATGTTGTGGATCGGC<br>GCTCACTGCCACACGAAAAT	1.5 μM
RUNX1	CATCGCTTTCAAGGTACTG<br>CATCCCAAGCTAGGAAGAC	1.5 μM
SF3B1	CTCTGTGTTGGCGGATAC<br>TTGTAGGTCTTGTGGATGAG	1.5 μM
SPG7	CCCATTTCCTGATTCTCTCT<br>GAGCACTGACCATCCATT	1.0 μM
UBASH3A	AACTGAACGTCTGATGCT<br>GTGTTGCTGTTATTGTTACC	1.0 μM
WLS	GCCTCAAACCCTCTGCTCTT<br>CAGGTGAGGCATAGAGGTGC	0.5 μM

WNT7A	AGAAGTCGCCCAACTACTGC<br>GCACGTGTTGCACTTGACAT	0.5 μM
IDH2	AAGCTGAAGAAGATGTGGAA<br>GCAGAGACAAGAGGATGG	1.0 μM

Table S3 Antibodies for FACS experiments

Antibody	Conjugates	Clone	Company	Catalog#
anti-human Lineage Cocktail (CD3, CD14, CD16, CD19, CD20, CD56)	BV510	OKT3; M5E2; 3G8; HIB19; 2H7; HCD56;	Biolegend	348807
CD34	APC	8G12	BD™	340441
CD38	PE-Cy7	HIT2	BD Pharmingen™	557945
CD45RA	FITC	HI100	BD Pharmingen™	561882
CD90	PerCP-Cy5.5	5E10	BD Pharmingen™	561557
CD49f	BV605	GoH3	BD OptiBuild™	740416
CD10	BV786	HI10a	BD Horizon™	564960
CD135	PE	4G8	BD Pharmingen™	558996
CD123	BV421	7G3	BD Horizon™	563362
IL1RAP	AF700	89412R	R&D	FAB6761N
CD45	PerCP-Cy5.5	HI30	BD Pharmingen™	564105
CD16	APC	B73.1	BD	5613

			Pharmingen™	04
CD56	APC	B159	BD	5555
			Pharmingen™	18
CD19	PE-Cy7	SJ25C1	BD	5609
			Pharmingen™	11
CD3	FITC	UCHT1	BD	5618
			Pharmingen™	06
CD14	APC-Cy7	MφP9	BD	5617
			Pharmingen™	09
CD10	PE	HI10a	BD	5610
			Pharmingen™	02
CD33	PE	WM53	BD	5618
			Pharmingen™	16

Material methods

Whole genome analysis

We assessed the quality of the raw sequencing data with FastQC v0.11.8 (https://www.bioinformatics.babraham.ac.uk/projects/fastqc/). Low-quality reads were removed by Trim Galore v0.4.4 using the default parameters (https://www.bioinformatics.babraham.ac.uk/projects/trim_galore/). High-quality reads were mapped to human reference genome (UCSC build version: hg38) using BWA v0.7.15 [1]. Thereafter, alignment results were processed according to the method described in the GATK best practice for detecting somatic mutation recommended by the Broad Institute [2]. Duplicated reads were marked with the *MarkDuplicates* module of GATK v4.1.4.1 [2]. Then we used Mutect2 of GATK v4.1.4.1 comparing each of the samples to the matched germline control by the default parameters for detection of somatic SNVs and indels [3]. In addition, copy number variations (CNVs) were estimated by the FACETS v0.5.14 package of R v3.6.3 [4]. Finally, ANNOVAR software was used to annotate the variation detected [5].

Clonal analysis

Using the Mutect2 output, variant allele frequency (VAF) for each mutation was calculated by the number of variant reads divided by total reads, and the VAFs of mutations in samples of different disease stages were used as input for PyClone analysis. PyClone is a tool based on Bayesian clustering, which is used to group sets somatic mutations into putative clonal clusters and estimate their cellular prevalence[6]. In this study, PyClone v0.13.1 analysis was used to infer the clonal composition and estimate the cancer cell fraction (CCF) of the patient P1001 in different disease stages with default parameters. To track the frequency of a SNV across disease progression, if a mutation was verified in at least one sample, regardless of individual sample validation results, the frequency of that mutation in other samples would be retained[7].

Single-cell targeted sequencing

For targeted sequencing, specific primers were designed against the target gene by Primer3. To amplify specific regions of target gene, we first performed specific target amplification of single

cell-genome amplification products using TaKaRa LA Taq® DNA Polymerase. All common sequence (CS) tagged (CS-F primer: 5′-ACACTGACGACATGGTTCTACA-3′, CS-R primer: 5′-TACGGTAGCAGAGACTTGGTCT-3′) specific primers were pooled and diluted to make a final concentration of 0.5-2 μM for each primer. The amplification PCR mix for each single cell was prepared as follows: 0.5 μl LA Taq® DNA Polymerase, 5 μl of 10× reaction buffer with $MgCl_2$, 8 μl dNTP mixture, 7.5 μl primer pool, and 4 μl WGA products. Then, PCR amplification was performed as follows: 94°C for 1 min; 2 cycles of 94°C for 30 s and 60°C for 4min; 35 cycles of 94°C for 30s and 50°C for 30s, 72°C for 30s. Then all the products of WGA were purified with Agencourt AMPure XP beads (Beckman Coulter) followed by barcoding PCR. In brief, 10nt-barcode modified from Barcode Sequences for Access Array™ Barcode Library for Illumina sequencer, tagged with CS-R primer, used in barcoding PCR. We performed barcoding PCR using TaKaRa LA Taq® DNA Polymerase according to the manufacturer's protocol. The products of barcoding PCR were pooled and purified, followed by library using VAHTSTM Universal DNA Library Prep Kit for Illumina® V3 (Vazyme). We assessed the quality of the barcoded samples with Qubit3.0 (Thermofisher) and 2100 Bioanalyzer (Agilent).

Supplemental references:

1 Li H, Durbin R. Fast and accurate long-read alignment with Burrows–Wheeler transform. Bioinformatics. 2010; 26(5): 589-95. https://doi.org/10.1093/bioinformatics/btp698.

2 McKenna A, Hanna M, Banks E, Sivachenko A, Cibulskis K, Kernytsky A, et al. The Genome Analysis Toolkit: a MapReduce framework for analyzing next-generation DNA sequencing data. Genome Res. 2010; 20(9): 1297-303. https://doi.org/10.1101/gr.107524.110.

3 Cibulskis K, Lawrence MS, Carter SL, Sivachenko A, Jaffe D, Sougnez C, et al. Sensitive detection of somatic point mutations in impure and heterogeneous cancer samples. Nat Biotechnol. 2013; 31(3): 213-9. https://doi.org/10.1038/nbt.2514.

4 Shen R, Seshan VE. FACETS: allele-specific copy number and clonal heterogeneity analysis tool for high-throughput DNA sequencing. Nucleic Acids Res. 2016; 44(16): e131. https://doi.org/10.1093/nar/gkw520.

5 Wang K, Li M, Hakonarson H. ANNOVAR: functional annotation of genetic variants from high-throughput sequencing data. Nucleic Acids Research. 2010; 38(16): e164-e. https://doi.org/10.1093/nar/gkq603.

6 Roth A, Khattra J, Yap D, Wan A, Laks E, Biele J, et al. PyClone: statistical inference of clonal population structure in cancer. Nat Methods. 2014; 11(4): 396-8. https://doi.org/10.1038/nmeth.2883.

7 Engle EK, Fisher DA, Miller CA, McLellan MD, Fulton RS, Moore DM, et al. Clonal evolution

revealed by whole genome sequencing in a case of primary myelofibrosis transformed to secondary acute myeloid leukemia. Leukemia. 2015; 29(4): 869-76. https://doi.org/10.1038/leu.2014.289.
